# Post-Anesthesia Recovery: A Comprehensive Review of Sampe, Modified Aldrete, and White Scoring Systems

**DOI:** 10.7759/cureus.70935

**Published:** 2024-10-06

**Authors:** Prachi P Deshmukh, Vivek Chakole

**Affiliations:** 1 Anaesthesiology, Jawaharlal Nehru Medical College, Datta Meghe Institute of Higher Education and Research, Wardha, IND

**Keywords:** modified aldrete scoring system, patient management, post-anesthesia recovery, recovery scoring systems, sampe scoring system, white scoring system

## Abstract

Post-anesthesia recovery is a vital phase in the perioperative continuum, where the quality of care and monitoring heavily influence patient outcomes. This comprehensive review examines the Sampe, Modified Aldrete, and White Scoring Systems, pivotal in evaluating patients' readiness for discharge from the Post-Anesthesia Care Unit (PACU). The review delves into the historical evolution of post-anesthesia care, highlighting the transition from minimal post-operative support to the establishment of PACUs and the subsequent development of structured recovery scoring systems. Each scoring system is analyzed in detail, focusing on its components, criteria, scoring methodology, advantages, and limitations. A comparative analysis underscores these systems' similarities and differences, sensitivity, specificity, and practical applications in clinical settings. Additionally, the review discusses the clinical implications of these scoring systems in enhancing patient management, improving safety, and ensuring standardized care. Emerging technologies and future directions in recovery assessment are also explored, providing insights into potential innovations. This review aims to equip healthcare professionals with a deeper understanding of these scoring systems, facilitating informed decisions to optimize post-anesthesia care and patient outcomes.

## Introduction and background

Post-anesthesia recovery is a critical phase in the perioperative process, encompassing the period immediately following the cessation of anesthesia until the patient regains sufficient physiological stability to be safely discharged from the post-anesthesia care unit (PACU) [1]. This phase is marked by restoring consciousness, normalizing vital signs, recovering motor and sensory functions, and alleviating any residual effects of anesthetic agents. Effective management of post-anesthesia recovery is essential to minimize complications, enhance patient comfort, and ensure optimal outcomes [2]. The assessment of patients during the post-anesthesia recovery period requires a systematic and objective approach to determine their readiness for discharge from the PACU. Recovery scoring systems play a pivotal role in this process by providing standardized criteria to evaluate the patient's recovery status. These systems help clinicians make informed decisions regarding the patient's readiness to transition from intensive monitoring to less intensive care environments, thereby improving patient safety and streamlining the discharge process [3].

Recovery scoring systems are designed to assess various physiological parameters, including consciousness level, respiratory status, cardiovascular stability, motor function, and pain control [4]. By quantifying these parameters, clinicians can objectively monitor the patient's progress, identify deviations from the expected recovery trajectory, and intervene promptly if necessary. Furthermore, standardized scoring systems facilitate clear communication among healthcare providers and ensure consistency in patient care [5]. This comprehensive review examines three widely used post-anesthesia recovery scoring systems: the Sampe Scoring System, the Modified Aldrete Scoring System, and the White Scoring System. This review aims to provide a detailed analysis of each system's components, criteria, and scoring methodology, highlighting their advantages and limitations. By comparing these scoring systems, the review seeks to elucidate their clinical applications, relevance, and impact on patient management during post-anesthesia recovery.

## Review

Historical background

Evolution of Post-Anesthesia Care

The evolution of post-anesthesia care over the past century reflects significant advancements in anesthetic techniques, surgical practices, and patient management. In the early era of anesthesia, post-operative care was minimal; patients often recovered without specialized monitoring or support. This lack of structured post-anesthesia care resulted in elevated rates of morbidity and mortality due to unrecognized complications, including respiratory depression, cardiovascular instability, and inadequate pain management [6]. The mid-20th century marked a paradigm shift in postoperative care by establishing Post-Anesthesia Care Units (PACUs), commonly called recovery rooms. These units were created to offer a controlled environment for closely monitoring patients during the immediate post-operative phase. The introduction of PACUs significantly enhanced patient outcomes by facilitating the early detection and intervention of post-anesthesia complications [7]. Over time, the scope of post-anesthesia care has broadened to encompass the management of physiological parameters and the provision of comprehensive care focused on enhancing patient comfort and promoting faster recovery. Innovations such as multimodal analgesia, advanced monitoring technologies, and individualized recovery plans have further refined the quality of care in the PACU. Today, post-anesthesia care is acknowledged as a vital component of the perioperative continuum, ensuring that patients transition safely from the effects of anesthesia to a stable post-operative state [8].

Development of Recovery Scoring Systems

The development of recovery scoring systems has closely followed the evolution of post-anesthesia care, addressing the necessity for standardized methods to assess and document patients' recovery progress. These systems were introduced to establish objective criteria for evaluating essential physiological and functional parameters, facilitating consistent and accurate assessments of recovery status [9]. One of the earliest and most influential recovery scoring systems is the Aldrete Score, developed by Dr. J. Aldrete in 1970. The Aldrete Score evaluates five critical parameters: activity, respiration, circulation, consciousness, and oxygen saturation. Each parameter is assigned a score ranging from 0 to 2, with a maximum possible score of 10. The simplicity and effectiveness of the Aldrete Score led to its widespread adoption, establishing a foundation for subsequent recovery scoring systems [10]. In response to the evolving needs of post-anesthesia care, the Modified Aldrete Scoring System was introduced to include additional parameters, such as pain and nausea/vomiting, reflecting a more comprehensive approach to recovery assessment. This modified version maintained the core principles of the original Aldrete Score while enhancing its applicability across a broader range of clinical scenarios [11]. Other notable recovery scoring systems, such as the Sampe Scoring System and the White Scoring System, have been developed to address specific aspects of post-anesthesia recovery. Each system has unique criteria and scoring methodology tailored to different patient populations and clinical settings. For example, the Sampe Scoring System emphasizes the early identification of recovery milestones, while the White Scoring System focuses on assessing cognitive and motor functions [12]. The ongoing refinement of recovery scoring systems reflects the dynamic nature of post-anesthesia care and the continuous efforts to enhance patient outcomes. These systems provide valuable tools for clinicians, enabling informed decision-making regarding patient discharge from the PACU and ensuring a smooth transition to the next phase of recovery [13].

Sampe scoring system

Description and Components

The SAMPE checklist is a novel tool designed to assess patient readiness for discharge from the Post-Anesthesia Care Unit (PACU) following surgery with anesthesia. Key features of the SAMPE checklist include its concise and straightforward administration and its practical evaluation of recovery dimensions. Developed through an extensive literature review and consensus among senior anesthesiologists and PACU nursing staff, the initial version of the SAMPE checklist addressed eight critical domains essential for safe discharge: hemodynamics, consciousness, breathing, oxygen saturation, pain, nausea/vomiting, bleeding, and motor function. The original checklist required a minimum score of 13 out of 16 for discharge, with certain conditions identified as contraindications [14]. To enhance adherence to the checklist, the SAMPE checklist underwent simplification by eliminating the necessity for a final cumulative score. All items were converted to a binary format (checked or not checked) rather than a graded scale. The same eight domains were preserved and deemed vital for assessing discharge readiness [15]. The revised SAMPE checklist evaluates the eight key recovery domains using a binary "yes/no" response for each criterion. Patients may be discharged if they meet acceptable standards across all eight criteria, eliminating the need for final score calculation. This streamlined approach aims to improve caregiver adherence compared to the original version, which required summing up a final score [16].

Advantages and Limitations

The SAMPE checklist presents several advantages over other assessment tools, such as the modified Aldrete and White scores, for evaluating post-anesthesia recovery and discharge readiness. It is a brief and user-friendly instrument that simplifies the discharge process by requiring all items to be checked for readiness rather than necessitating the summation of a final score. This binary format (checked or not checked) enhances adherence to the checklist compared to the original version [14]. The SAMPE checklist retains the same critical domains as its predecessor, deemed essential for safe discharge. In a study involving nearly 1,000 patients, the SAMPE checklist demonstrated satisfactory agreement with the White score, although it showed lower agreement with the modified Aldrete score. This indicates that the SAMPE checklist provides a practical assessment of key recovery dimensions [14]. Despite its advantages, the SAMPE checklist also has limitations. It assesses only the presence or absence of recovery criteria, lacking an evaluation of the quality or degree of recovery, as is the case with the modified Aldrete and White scores. Furthermore, the checklist requires additional validation in more diverse patient populations to establish reliability and generalizability. The binary nature of the checklist may oversimplify the assessment of complex recovery parameters [17].

Clinical Applications and Relevance

The SAMPE checklist is an innovative tool for assessing patient readiness for discharge from the Post-Anesthesia Care Unit (PACU), offering several advantages over the modified Aldrete and White scoring systems. One of its primary strengths is its simplicity and ease of use. The SAMPE checklist is concise and straightforward to administer, enhancing the likelihood of caregivers' adherence. Transforming all items into a binary format (checked or not checked) eliminates the need for summing a final score. This streamlined approach can help standardize the discharge process and improve compliance with the assessment criteria [14]. Regarding comprehensive recovery assessment, the SAMPE checklist evaluates eight key critical domains for safe discharge: hemodynamics, consciousness, breathing, oxygen saturation, pain, nausea/vomiting, bleeding, and motor function. This provides a more thorough assessment than the modified Aldrete score, which only considers five parameters. Additionally, the SAMPE checklist includes evaluations for pain and postoperative nausea and vomiting (PONV) akin to the White score but in a simpler format [14]. Research has demonstrated that the SAMPE checklist exhibits satisfactory agreement with the White score and lower agreement with the modified Aldrete score. This suggests that it aligns reasonably well with established tools while being easier to use. Moreover, the conservative requirement that all criteria must be met before discharge ensures that patients are fully prepared to leave the PACU, potentially improving postoperative outcomes and reducing complication rates [14].

Modified Aldrete scoring system

Description and Components

The modified Aldrete scoring system is widely used to evaluate patients' readiness for discharge from the post-anesthesia care unit (PACU) following surgery. This scoring system assesses five key criteria to determine the patient's recovery status. The first criterion is activity, which evaluates patients' ability to move their extremities voluntarily or upon command. Patients can receive 2 points for being able to move all four extremities, 1 point for moving two extremities, and 0 points if they cannot move any extremities [18]. The second criterion is respiration, which assesses the patient's breathing ability. A patient scores 2 points if they can breathe deeply and cough freely, 1 point if they exhibit limited breathing or dyspnea, and 0 points if they are apneic. The third criterion is circulation, which compares the patient's systemic blood pressure to their pre-anesthetic level. Patients can score 2 points if their blood pressure is within 20% of the pre-anesthetic level, 1 point between 20% and 49% of the pre-anesthetic level, and 0 points if it exceeds 49% of the pre-anesthetic level [19]. The fourth criterion is consciousness, which assesses the patient's level of alertness. Patients can score 2 points if they are fully awake, 1 point if arousable upon calling, and 0 points if unresponsive. The final criterion is oxygen saturation, which measures the patient’s SpO2 level. A patient can score 2 points if their SpO2 is greater than 92% on room air, 1 point above 90% with supplemental oxygen, and 0 points if it falls below 90% even with supplemental oxygen [20]. The scores from each category are summed to produce a total modified Aldrete score, which ranges from 0 to 10. A score of 9-10 generally indicates that the patient is ready for discharge from the PACU. In contrast, a score lower than nine typically suggests that the patient requires further monitoring and management in the PACU. Overall, the modified Aldrete scoring system objectively assesses the patient's recovery status, guiding the decision for safe discharge from the PACU [21].

Comparison With the Original Aldrete Score

The modified Aldrete scoring system is an updated version of the original Aldrete scoring system, which was developed in 1970 by Jorge Antonio Aldrete. The original system assessed five criteria: activity, respiration, circulation, consciousness, and color/oxygen saturation. At that time, skin coloration was used as a surrogate marker for oxygen saturation before the advent of pulse oximetry [10]. Introduced in 1995, the modified Aldrete scoring system retains the evaluation of the same five criteria: activity, respiration, circulation, consciousness, and oxygen saturation. However, it notably replaces the assessment of skin coloration with pulse oximetry, allowing for direct measurement of oxygen saturation (SpO2). This change enhances the objectivity and accuracy of assessing the patient’s oxygenation status, as opposed to the more subjective evaluation of skin color used in the original system [18]. In the original and modified Aldrete scoring systems, a score of 9 or greater is deemed adequate for discharge from the post-anesthesia care unit (PACU). The modified Aldrete scoring system is now more widely adopted due to its comprehensive and reliable assessment of a patient’s recovery status following anesthesia or procedural sedation. The incorporation of pulse oximetry has significantly improved the accuracy and consistency of the scoring system compared to its predecessor [22].

Advantages and Limitations

The modified Aldrete scoring system is a widely recognized tool for evaluating patients' readiness for discharge from the post-anesthesia care unit (PACU) following surgery. This scoring system assesses five key criteria: activity, respiration, circulation, consciousness, and oxygen saturation. A notable advantage of the modified Aldrete scoring system is its incorporation of pulse oximetry for measuring oxygen saturation, which offers a more objective and accurate assessment than the original system's reliance on skin coloration. This enhancement helps standardize discharge criteria, ensuring patients are only discharged when fully recovered and deemed safe to leave the PACU, thereby improving patient safety [23]. Moreover, the comprehensive evaluation of the five criteria provides a structured and systematic approach to assessing a patient’s recovery status. This reduces the risk of prematurely discharging patients who have not yet fully recovered, which is essential for ensuring their safety and well-being. However, the modified Aldrete scoring system is not without limitations. One significant drawback is its lack of direct assessment of pain, a common postoperative complication. Additionally, certain system components, such as consciousness, may still be subjective and influenced by the observer's judgment [24]. Furthermore, the system does not consider specific conditions, such as administering certain medications (e.g., beta-blockers) or the presence of pain or nausea, which could impact the patient’s recovery. It also omits assessments for bleeding, nausea, vomiting, or urine output, important parameters in some surgical procedures. Despite these limitations, the modified Aldrete scoring system remains a widely used and effective tool for assessing patient readiness for discharge from the PACU. Its objective measurement of oxygen saturation, combined with a comprehensive evaluation of other critical criteria, makes it an invaluable instrument in postoperative recovery [25].

Clinical Applications and Relevance

The modified Aldrete scoring system is a widely utilized tool in clinical settings for assessing patients' readiness for discharge from the post-anesthesia care unit (PACU). Its primary purpose is to determine when a patient is ready to transition from the PACU to the next stage of recovery, whether that be a hospital ward, an ambulatory care unit, or home. By offering a standardized and objective evaluation of a patient’s recovery status, the system ensures that patients are discharged only when fully recovered and deemed safe to leave the PACU. This structured and systematic approach helps standardize discharge criteria, minimizing the risk of premature or inappropriate discharges [26]. The modified Aldrete scoring system is particularly pertinent for ambulatory or outpatient surgical procedures, where patients are expected to be discharged shortly after the operation. It assists in determining whether a patient is suitable for home recovery, ensuring they are stable and capable of managing their post-operative care. Additionally, the scoring system allows clinicians to closely monitor a patient’s progress through various stages of recovery, facilitating the early identification of any issues or complications that may necessitate further intervention. This ongoing monitoring ensures that patients receive the appropriate level of care and that potential complications are addressed promptly [21]. Furthermore, the modified Aldrete scoring system enhances communication during handoffs between care providers, ensuring that critical information about a patient's status is effectively conveyed during care transitions. This is especially important in the PACU, where patients are often transferred from the operating room to the PACU and subsequently to the next level of care. By providing a standardized scoring system, the modified Aldrete system ensures that all care providers clearly understand a patient’s condition, reducing the risk of errors and ultimately improving patient outcomes. Overall, the modified Aldrete scoring system is a clinically relevant and widely adopted tool that supports safe and efficient patient care throughout the continuum from the operating room to the next stage of recovery [27-29].

White scoring system

Description and Components

The White scoring system, introduced in 1999, is a comprehensive tool for evaluating patients' readiness for discharge from the post-anesthesia care unit (PACU). This system assesses seven critical parameters: consciousness, activity, circulation, respiration, oxygen saturation, pain, and emesis (nausea/vomiting) [22]. Each criterion is scored on a scale of 0 to 2, yielding a total possible score of 14. To be deemed ready for discharge, patients must attain a minimum score of 12, with no individual score falling below 1. This requirement ensures that all facets of the patient's recovery are thoroughly addressed before transitioning to the next level of care [28]. The specific scoring for each criterion is outlined as follows: Consciousness is assessed based on the patient's level of wakefulness, awarding 2 points for being fully awake, 1 point for being arousable, and 0 points for unresponsiveness. Activity evaluates the patient's ability to move their extremities, with 2 points for moving all extremities, 1 point for moving two extremities, and 0 points for an inability to move. Circulation is assessed based on the patient's blood pressure at the pre-anesthetic level, granting 2 points for a deviation within 20%, 1 point for a deviation of 20-50%, and 0 points for a deviation exceeding 50%. Respiration is scored on the patient's ability to breathe deeply, awarding 2 points for deep breathing, 1 for shallow or dyspneic breathing, and 0 for apneic status [30]. Oxygen saturation is evaluated with 2 points for saturation levels of ≥ 92% on room air, 1 point for ≥ 92% with supplemental oxygen, and 0 points for < 92% with supplemental oxygen. Pain is assessed according to the patient's level of discomfort, with 2 points for minimal pain, 1 point for moderate pain, and 0 points for severe pain. Lastly, emesis is scored with 2 points for no nausea or vomiting, 1 for nausea only, and 0 for active vomiting [30].

Advantages and Limitations

The White scoring system, introduced by White in 1999, is a comprehensive tool designed to assess patients' readiness for discharge from the post-anesthesia care unit (PACU). This system evaluates seven critical parameters: consciousness, activity, circulation, respiration, oxygen saturation, pain, and emesis (nausea/vomiting) [13]. Each criterion is scored on a scale of 0 to 2, resulting in a total possible score of 14. To be considered ready for discharge, a patient must achieve a minimum score of 12, with no individual score falling below 1. This scoring methodology offers a more thorough evaluation of a patient's recovery status compared to simpler systems like the modified Aldrete score [31]. One of the primary advantages of the White scoring system is its comprehensive nature. By incorporating parameters such as pain and nausea/vomiting, the system ensures that patients are stable and ready for safe discharge, as these factors are critical to postoperative recovery. Research has demonstrated that the White scoring system is superior to the modified Aldrete system in facilitating safe PACU discharge [32]. However, the complexity of the White scoring system may also pose a limitation. Evaluating seven criteria on a 0-2 scale results in a more intricate assessment than the modified Aldrete's five-parameter system. This complexity may render the White scoring system less practical in certain clinical settings [33]. Despite its advantages, the White scoring system has not gained the same widespread adoption as the modified Aldrete system. The familiarity and simplicity of the modified Aldrete score may contribute to its continued use in many institutions, even though the White system provides a more comprehensive approach to assessing post-anesthesia recovery [33].

Clinical Applications and Relevance

The White scoring system, commonly called the "fast-track criteria," has several clinical applications, particularly concerning discharge from the post-anesthesia care unit (PACU). One of its primary applications is its comprehensive assessment capability. Unlike other scoring systems, the White scoring system evaluates a broader range of parameters, including pain, nausea, and vomiting. This holistic approach enhances patient safety by addressing common postoperative complications that may be overlooked by simpler scoring systems [34]. Research indicates that the White scoring system is superior to the modified Aldrete scoring system in ensuring safe discharge from the PACU. Its wider scope and higher minimum score requirement enhance its effectiveness in accurately determining a patient’s readiness for discharge [33]. Additionally, the White scoring system is relevant as a "fast-track criterion," as it aims to identify patients who can be directly transferred from the operating room to Phase II recovery, thereby reducing the need for Phase I in the PACU. This can improve patient flow and resource utilization by allowing patients to bypass the initial PACU phase [35]. Regarding patient safety, the White scoring system's comprehensive assessment and elevated minimum score requirement are critical in confirming that patients are stable and ready for safe discharge, thereby minimizing the risk of complications and readmissions. Moreover, its facilitation of direct transfer to Phase II recovery streamlines patient care, enhancing overall efficiency and resource use [36]. The White scoring system is also highly pertinent in clinical practice, providing a structured and thorough tool for evaluating postoperative recovery. This is essential for ensuring patient safety and optimizing care. Its ability to comprehensively assess a patient’s readiness for discharge makes it an invaluable resource for healthcare professionals working in the PACU [22].

Comparative analysis

Key Similarities and Differences

The Sampe, Modified Aldrete, and White scoring systems all assess patient readiness for discharge from the post-anesthesia care unit (PACU). However, they exhibit several key differences. One significant distinction lies in the specific domains and criteria each system evaluates. The Sampe checklist assesses eight key domains using a binary pass/fail scale, requiring full recovery across all areas before a patient can be discharged. In contrast, the Modified Aldrete scoring system evaluates five criteria, activity, respiration, circulation, consciousness, and oxygen saturation, using a numerical score ranging from 0 to 2 for each criterion, with a total score of 8 or higher required for discharge. The White scoring system enhances the Aldrete criteria by incorporating additional assessments for pain and nausea/vomiting [14]. The scoring methodologies themselves also differ markedly. The Sampe checklist employs a simple binary approach, providing no final numerical score. The Modified Aldrete system yields a total score of 10, while the White system mandates a minimum overall score of 12, with no individual score below 1 [14]. Regarding conservatism regarding discharge decisions, the Sampe checklist has been found to be the most conservative, denying discharge to 26% of patients who would have qualified under the White or Aldrete systems criteria. The Modified Aldrete system is generally less conservative than the Sampe checklist, while the White system occupies a middle ground [14]. Finally, the systems differ in terms of adherence and ease of use. The Sampe checklist was designed to be user-friendly and improve documentation through its binary approach. The Modified Aldrete system, although widely utilized, can be more labor-intensive to complete, which may compromise adherence. Like the Sampe checklist, the White scoring system was also developed to emphasize ease of use and identify patients who can safely bypass the PACU [14].

Sensitivity and Specificity of Each System

The Sampe checklist has been evaluated for its sensitivity and specificity in assessing patient readiness for discharge. The results demonstrate that the Sampe checklist has a sensitivity of 65.5% (95% CI: 60.7-70.1) and a specificity of 72.6% (95% CI: 66.4-78.3) in identifying frail or prefrail individuals when compared to the Fried frailty phenotype. This suggests that the Sampe checklist possesses a moderate ability to accurately identify patients ready for discharge while being cautious enough to deny discharge to many patients who may not be fully recovered [37]. In contrast, the available data did not detail specific sensitivity and specificity values for the Modified Aldrete scoring system. However, it is noted that the Sampe checklist exhibited lower agreement with the Modified Aldrete system compared to the White scoring system. This indicates that the Sampe checklist may adopt a more conservative stance in assessing patient readiness for discharge relative to the Modified Aldrete system [14]. Similarly, the search results do not present direct comparisons of sensitivity and specificity between the White fast-track scoring system and the other two systems. It is mentioned that the Sampe checklist demonstrated satisfactory agreement with the White scoring system, suggesting that the White system may align more closely with the Sampe checklist in its evaluation of discharge readiness. However, the specific sensitivity and specificity figures for the White system remain undefined [33]. Overall, the information indicates that the Sampe checklist is a more conservative tool, exhibiting moderate sensitivity and specificity, while the comparative performance of the Modified Aldrete and White scoring systems lacks clear delineation in the available data. Further research is warranted to provide a more comprehensive understanding of the relative strengths and limitations of these various post-anesthesia recovery assessment tools [18].

Ease of Use and Practicality in Clinical Settings

The Sampe, Modified Aldrete, and White scoring systems are all utilized to assess patient readiness for discharge from the post-anesthesia care unit (PACU) following surgery. The Sampe checklist is a novel recovery room discharge tool designed to offer a practical and user-friendly assessment of a patient’s recovery status. Using a binary scale, it evaluates eight key domains, including consciousness, respiratory status, circulation, pain, and surgical bleeding. For a patient to be deemed ready for discharge, they must meet all criteria. This checklist aims to simplify and enhance adherence and documentation by PACU staff compared to earlier scoring systems [38]. The Modified Aldrete scoring system, a widely employed tool, assesses five critical criteria: activity, respiration, circulation, consciousness, and oxygen saturation. Each criterion is scored from 0 to 2, yielding a maximum total score of 10. Generally, a score of 8 or higher is considered the threshold for PACU discharge. In contrast, the White fast-track scoring system was developed as an alternative to the Modified Aldrete system to more effectively identify patients who can bypass the PACU and be discharged directly to the ward or home. Besides the Aldrete criteria, the White system also evaluates pain and nausea/vomiting. To qualify for fast-tracking, patients must achieve specific thresholds across all parameters [18]. Comparative studies indicate that the Sampe checklist demonstrates satisfactory agreement with the White scoring system but shows lower agreement with the Modified Aldrete system. Notably, the Sampe checklist is more conservative, as it avoids discharge for 26% of patients who meet the White or Aldrete score criteria. The primary distinctions lie in the Sampe checklist employing a binary pass/fail approach for each domain rather than a graded scoring system, and it mandates complete recovery across all domains before discharge [14].

Clinical implications

Impact on Patient Management and Recovery

The Sampe checklist, Modified Aldrete, and White scoring systems significantly impact patient management and recovery in the post-anesthesia care unit (PACU). Employing a more comprehensive discharge tool, such as the Sampe checklist or the White scoring system, can lead to safer discharge decisions by ensuring patients meet a broader set of criteria before leaving the PACU [14]. The traditional Aldrete system does not evaluate critical issues like pain and nausea/vomiting, which are common postoperative concerns. In contrast, the Sampe checklist assesses eight key domains: hemodynamics, consciousness, breathing, oxygen saturation, pain, nausea/vomiting, bleeding, and motor blockade. The White fast-track scoring system similarly incorporates the Aldrete criteria while addressing pain and nausea/vomiting, offering advantages in determining whether patients are suitable for bypassing the PACU [14]. Implementing the Sampe checklist or White scoring system could potentially reduce PACU's length of stay and enhance operating room efficiency by facilitating the faster discharge of patients who meet the more comprehensive recovery criteria. Research indicates that the White scoring system is superior to the Aldrete system in assessing the appropriateness of directly discharging patients to Phase II recovery [14]. The transition of patients from the PACU to the ward or home discharge is a critical handoff point, where adverse events may occur if recovery is not adequately evaluated. Utilizing a more robust discharge tool like the Sampe checklist or White scoring system can help maintain continuity of care, minimize errors, and prevent complications by ensuring patients are fully prepared for the next level of care [39].

Guidelines for Selecting an Appropriate Scoring System

Several key guidelines should be considered when selecting an appropriate scoring system for assessing post-anesthesia recovery and determining discharge readiness. The scoring system must comprehensively evaluate a wide range of recovery factors, including vital signs, activity level, consciousness, respiration, oxygen saturation, pain, nausea/vomiting, bleeding, and urine output. Systems that assess only a limited number of criteria, such as the original Aldrete system, may be inadequate, especially for fast-tracking patients after ambulatory surgery [40]. Moreover, the scoring system should align with current anesthesia and surgical techniques, particularly those utilizing shorter-acting anesthetics and minimally invasive procedures that facilitate faster recovery. Systems developed decades ago may require updates to reflect modern practices and patient expectations. Ease of use and standardization are also critical considerations. The scoring system should be straightforward, concise, and easy to administer to promote consistent application by clinicians. Standardized scoring systems enhance communication and continuity of care as patients transition between different units and providers [41]. The scoring system must be validated to accurately predict when patients can be safely discharged from the post-anesthesia care unit (PACU), whether to a hospital ward, a phase II recovery area, or home. The system should minimize the risk of premature discharge by ensuring patients meet well-defined criteria concerning vital signs, pain control, nausea/vomiting, and ambulation ability. Finally, the scoring system must be suitable for the specific patient population being assessed, whether pediatric, geriatric, or ambulatory surgery patients, as these groups may have unique recovery needs. Scoring systems such as the White fast-track criteria, developed specifically for ambulatory surgery patients, may be particularly advantageous [39].

Role in Standardized Post-Anesthesia Care

The Sampe checklist, modified Aldrete, and White scoring systems play significant roles in standardizing post-anesthesia care and ensuring the safe discharge of patients from the post-anesthesia care unit (PACU). A key advantage of the Sampe checklist and White's scoring system is their more comprehensive assessment of recovery factors compared to the traditional Aldrete system. While the Aldrete system evaluates limited criteria such as activity, respiration, and circulation, Sampe and White's tools incorporate a broader range of measures, including pain, nausea/vomiting, bleeding, and motor function. This more robust evaluation helps patients meet well-defined criteria for safe discharge from the PACU [12]. Implementing the Sampe checklist or White's scoring system can improve discharge criteria and enhance decision-making. These standardized, validated tools provide clear guidelines for determining when patients are ready to transition out of the PACU, thereby reducing the risk of premature discharge that may occur with the more limited Aldrete system. Adopting these newer scoring systems helps align PACU discharge practices with contemporary anesthesia techniques and patient recovery expectations [42]. Furthermore, standardized scoring systems like the Sampe checklist and White's system enhance continuity of care as patients move between different units and providers. A common assessment framework facilitates better communication and handoffs, crucial during the critical transition from the PACU to the ward or home discharge. This approach helps minimize the risk of adverse events during this important handoff process [43].

Recent advances and research

Innovations in Post-Anesthesia Recovery Assessment

The development of the SAMPE checklist signifies a significant advancement in the assessment of post-anesthesia recovery. This innovative discharge tool was designed to provide a practical and comprehensive evaluation of recovery dimensions, addressing the limitations of existing scoring systems such as the Aldrete and White methods. The SAMPE checklist evaluates eight critical criteria: hemodynamics, consciousness, breathing, oxygen saturation, pain, nausea/vomiting, bleeding, and motor function. Studies have demonstrated that the SAMPE checklist exhibits satisfactory agreement with the White scoring system while showing lower agreement with the modified Aldrete score, suggesting potential advantages over the traditional Aldrete approach. Importantly, the SAMPE checklist is structured to be user-friendly, utilizing a binary yes/no format that may enhance adherence among healthcare staff compared to more complex scoring systems [14]. Although the modified Aldrete scoring system remains the most widely used tool for assessing post-anesthesia recovery, research has revealed its limitations in adequately evaluating patients for fast-tracking, particularly due to the lack of assessment for pain and nausea/vomiting. This is a significant shortcoming, as these symptoms are known to persist longer than other recovery parameters. Furthermore, existing scoring systems can be labor-intensive, which may hinder adherence among busy healthcare professionals in the post-operative environment [44]. Ongoing research is focused on identifying the most critical variables for assessing safe discharge from the post-anesthesia care unit and developing new instruments that are both efficient and user-friendly while maintaining reliability. Further validation studies of the SAMPE checklist in larger and more diverse patient populations will be essential for establishing its role as a viable alternative to existing post-anesthesia recovery assessment tools. Overall, the innovations embodied by the SAMPE checklist and continued research efforts aim to optimize the evaluation of patient discharge readiness, thereby enhancing post-operative care quality and safety [14].

Emerging Technologies and Methodologies

In recent years, significant advancements have been witnessed in post-anesthesia recovery assessment, with one notable innovation being the development of the SAMPE checklist. This new recovery room discharge tool is designed to evaluate various recovery dimensions practically. The SAMPE checklist assesses eight critical criteria: hemodynamics, consciousness, breathing, oxygen saturation, pain, nausea/vomiting, bleeding, and motor function. Research indicates that the SAMPE checklist demonstrates satisfactory agreement with the White scoring system while showing lower agreement with the modified Aldrete score. Its straightforward application and potential for high adherence among staff make it a promising option for formalizing PACU discharge [45]. Despite the widespread use of the modified Aldrete scoring system, it has been deemed inadequate for fast-tracking patients, particularly due to its failure to assess pain and nausea/vomiting. Additionally, existing tools can be labor-intensive to complete, which may compromise adherence among healthcare professionals. Researchers focus on identifying the most critical variables for evaluating safe PACU discharge and developing user-friendly instruments that maintain reliability. Further studies are necessary to validate the SAMPE checklist and to compare its performance against other scoring systems in larger, more diverse patient populations [33].

Future directions for scoring systems

A key development area in post-anesthesia recovery assessment is integrating more granular data into scoring systems. This approach could incorporate temporal and molecular data to provide personalized severity scores that guide individual care decisions. Additionally, there is potential to expand scoring systems to predict longer-term outcomes, such as post-discharge survival rates, thereby enhancing patient management and resource allocation efforts [46]. Another important direction is the creation of new scoring systems. For example, the SAMPE checklist is a recently introduced recovery room discharge tool that evaluates eight criteria: hemodynamics, consciousness, breathing, oxygen saturation, pain, nausea/vomiting, bleeding, and motor function. This innovative system aims to address the limitations of existing scoring tools by offering a practical assessment of recovery dimensions [14]. In parallel with developing new scoring systems, ongoing efforts exist to enhance existing tools. For instance, the widely used modified Aldrete scoring system could be improved by incorporating assessments of pain and nausea/vomiting, which are currently absent but essential for adequately evaluating patient suitability for fast-tracking [13]. A sustained focus on research and validation supports these advancements. Researchers are actively identifying the most critical variables for assessing safe discharge from the post-anesthesia care unit (PACU) and developing comprehensive and user-friendly instruments. Further studies are necessary to validate new scoring systems, such as the SAMPE checklist, and to compare their performance against existing tools in larger and more diverse patient populations [47].

## Conclusions

In conclusion, the evolution of post-anesthesia care and the development of recovery scoring systems have significantly enhanced the safety and efficacy of patient management during the critical post-operative period. From establishing Post-Anesthesia Care Units to introducing standardized scoring systems like the Aldrete, Modified Aldrete, Sampe, and White Scoring Systems, these advancements have provided clinicians with valuable tools to objectively assess and monitor recovery. Each scoring system offers unique advantages and addresses specific clinical needs, contributing to improved patient outcomes through early detection of complications and timely interventions. By understanding the strengths and limitations of each system, healthcare professionals can select the most appropriate tools for their practice, ultimately ensuring that patients receive the highest standard of care as they transition from anesthesia to recovery. This comprehensive review underscores the importance of continued innovation and research in post-anesthesia recovery assessment, paving the way for future patient safety and care quality enhancements.
